# Implementation of a program for treatment of acute infections in nursing homes without hospital transfer

**DOI:** 10.3389/fmed.2024.1333523

**Published:** 2024-05-20

**Authors:** Nadya Kagansky, Reena Rosenberg, Estela Derazne, Evelina Mazurez, Yochai Levy, Micha Barchana

**Affiliations:** ^1^Clalit Health Services, Tel Aviv, Israel; ^2^Shmuel Harofe Geriatric Medical Center, Beer Ya’akov, Israel; ^3^Tel Aviv University School of Medicine, Ramat Aviv, Israel; ^4^Rabin Medical Center, Beilinson Hospital, Petah-Tikva, Israel; ^5^Technion University School of Public Health, Haifa, Israel

**Keywords:** acute infections, hospitalization, nursing homes, intravenous, training program

## Abstract

**Background:**

Nursing care residents have high hospitalization rates. To address this, we established a unique virtual geriatric unit that has developed a program aimed at providing support to nursing homes.

**Aims:**

We aimed to evaluate effectiveness of in-house intravenous antibiotic treatment in nursing hospitals after the implementation of the specially designed training program.

**Methods:**

A cohort study of nursing home residents to evaluate a training program for providers, designed to increase awareness and give practical tools for in-house treatment of acute infections. Data obtained included types of infections, antibiotics used, hospital transfer, and length of treatment. Primary outcomes were in-house recovery, hospitalization and mortality. Univariate analysis and multivariable logistic regression analysis to assess association between different factors and recovery.

**Results:**

A total of 890 cases of acute infections were treated with intravenous antibiotics across 10 nursing homes over a total of 4,436 days. Of these cases, 34.8% were aged 90 years or older. Acute pneumonia was the most prevalent infection accounted for 354 cases (40.6%), followed by urinary tract infections (35.7%), and fever of presumed bacterial infection (17.1%). The mean duration of intravenous antibiotic treatment was 5.09 ± 3.86 days. Of the total cases, 800 (91.8%) recovered, 62 (7.1%) required hospitalization and nine (1.0%) resulted in mortality. There was no significant difference observed in recovery rates across different types of infections.

**Discussion:**

Appling a simple yet unique intervention program has led to more “in-house” residents receiving treatment, with positive clinical results.

**Conclusion:**

Treating in-house nursing home residents with acute infections resulted in high recovery rates. Special education programs and collaboration between healthcare organizations can improve treatment outcomes and decrease the burden on the healthcare system.

## Introduction

Hospitalization rates and recurrent hospitalization rates are significantly higher in older patients compared to the general adult population (1). Nursing care residents, experience higher hospitalization rates, often due to acute infections (2, 3). The relatively crowded living conditions in the nursing homes (NH) and treatment by the same healthcare providers increase the risk of contagion spread (4, 5). The residents usually suffer from multiple co-morbidities and are prescribed numerous medications (6, 7). The most common infections are urinary tract and respiratory tract, including aspiration pneumonia (2, 8). Proper management of acute infections is a crucial aspect of providing high-quality care to nursing facility residents (9, 10). Literature reports that approximately 40% of patients over 65 with acute infections require hospitalization for treatment (11, 12). Hospitalization of older adults increases negative outcomes such as acute delirium during the hospital stay, cognitive decline, decubitus ulcers, falls, nosocomial infections, and difficulty in returning to previous levels of functioning (13–15). Reducing these negative outcomes can probably be achieved by improving NH in-house treatment. Enabling intravenous treatment may be an important step in that direction. To address this a designated program was designed and executed at the central district of Clalit medical service, the largest mandated health service organization in Israel. Clalit Health Services is the major insurance organization in Israel, with 4,394,000 members. Among the insured, 13.52% are aged 65 or older (16). In the Central Region 15.71% of insured are aged 65 or older with approximately 2,800 residing in skilled nursing care facilities. High quality management of older patients living in long-term care facilities is one of the important aims of older adults’ care. To address this need, we established a unique virtual nursing home center (VNHC) with qualified geriatric professionals. One of the primarily goals of the unit was to improve treatment of acute infections.

Providers in nursing care facilities frequently face the challenging decision whether to treat residents with acute infections in-house or to hospitalize them (17, 18). The decision is complex and depends on various factors. In Israel, skilled nursing facilities are managed and regulated by the Health Ministry, while each resident continues to be insured by their health maintenance organization (HMO). The provisions of medications and of medical services outside of the facility are therefore the responsibility of the HMO. One crucial factor in Israel is the ability of the facility to provide intravenous (IV) antibiotics according to the health ministry criteria and whether the facility has been licensed by the health department to administer that treatment. However, despite being licensed, many facilities still prefer to hospitalize patients with acute infections. We observed other factors influencing this decision. Firstly, there is the additional workload imposed on the medical and nursing staff. Secondly, bureaucratic and logistic difficulties arise in obtaining the appropriate antibiotics. Furthermore, the inertia presents in many facilities with long-standing routines and protocols makes changes difficult to accomplish (19). Lastly, there is a lack of awareness about the complications of hospitalization for older adult patients. The program implemented was designed to address these factors in order to improve in-house quality of infection care. The aims of this study were to evaluate the positive and negative clinical outcomes of the program on in-house intravenous antibiotic treatment in the participating NH.

## Materials and methods

In an effort to improve quality of care for residents with acute infections and to prevent hospitalizations, our VNHC staff developed a program to train and provide support to nursing care staff in facilities specially focusing on treatment of acute infections with IV antibiotics. The program included improving the logistics and coordination between the NH and the HMO to facilitate efficient in-house treatment.

Skilled nursing care facilities within the Clalit Health Services in the Central District of Israel were contacted to assess their compliance with the Health Ministry’s criteria for administering IV treatment and whether they had obtained the necessary approval. Out of 26 facilities, only 10 met the criteria. Surprisingly, a review of our HMO database showed high hospitalizations rates (34%) and low IV antibiotics order rates (5%) at these facilities. A 3-pronged intervention was developed by the VNHC staff to aid facilities in providing IV antibiotic treatment. Usually, NH personal receives lower quality of education compared to the hospital staff. Therefore, firstly experienced specialist in geriatric medicine provided lectures to the providers, aiming to increase their awareness and knowledge of hospital complications on older adults’ patients. The providers staff were encouraged to enroll in a designated course on infection diseases treatment in older adult patients. These educational steps helped enhance the knowledge of NH staff and created stronger connections between the healthcare organization’s staff and NH staff. Secondly, enhanced support system was given to the providers. Telephone consultations by the VNHC staff, including senior physician, geriatric expert practitioner nurse and pharmacological staff were made available to the nursing home providers. Thirdly, the logistics of ordering and transporting the required IV antibiotics were streamlined to allow for rapid delivery of the necessary treatments. Ultimately, the decision on how to treat each patient was entrusted to the nursing home staff. While all decisions were made by an in-house physician, a geriatric specialist was always a geriatric specialist on call as needed.

Over a four-year period (2012–2016) following the initiation of the intervention in 2012, the VNHC chief physician kept a register of data on each resident who was treated for an acute infection requiring IV antibiotics in the 10 facilities. All residents met the criteria of the Health Ministry for nursing care or mental frailty, including dependence on others for activities of daily living such as mobility, eating, bathing, getting dressed, toileting, transferring, and continence or requiring continues supervision because of advanced dementia

The data was obtained from the electronic medical records (EMR) of each patient. Data collected included age, gender, name of nursing home facility (NHF); for each acute infection, we recorded the diagnosis, date of diagnosis, date of initiation and completion of treatment, and type of antibiotic given. Recovery or a positive outcome were defined as completing antibiotic course and recovery from symptoms within the NHF while hospital transfer or death were defined as negative outcomes.

### Statistical analysis

**Figure 1:**
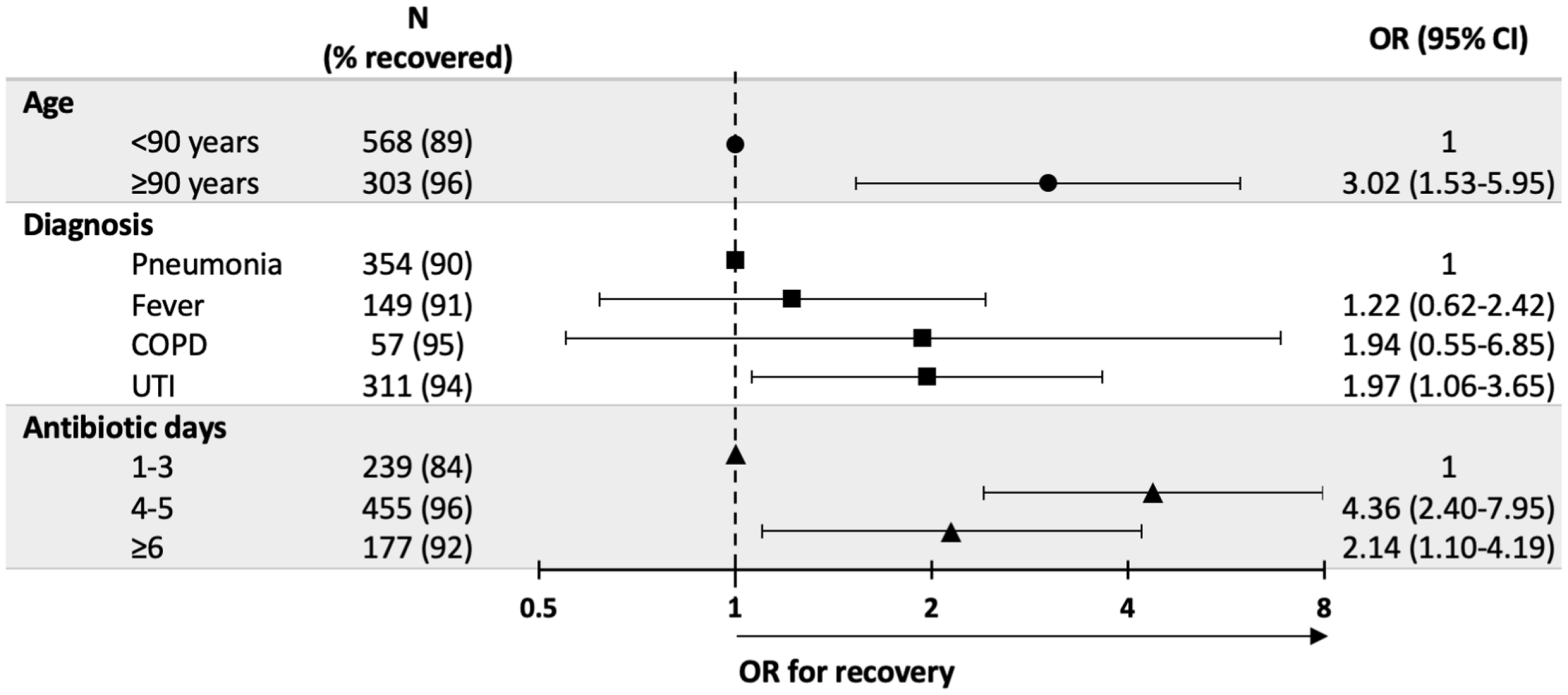
Factors associated with recovery–results from multivariable logistic regression*. *Variables included in the model: diagnosis, patient sex and age, antibiotic type, days of antibiotic treatment, nursing homes type and year of treatment. Variables not statistically significant were not included in figure. UTI, urinary tract infection, COPD, chronic obstructive pulmonary disease, OR, Odds Ratio.

**Figure 2:**
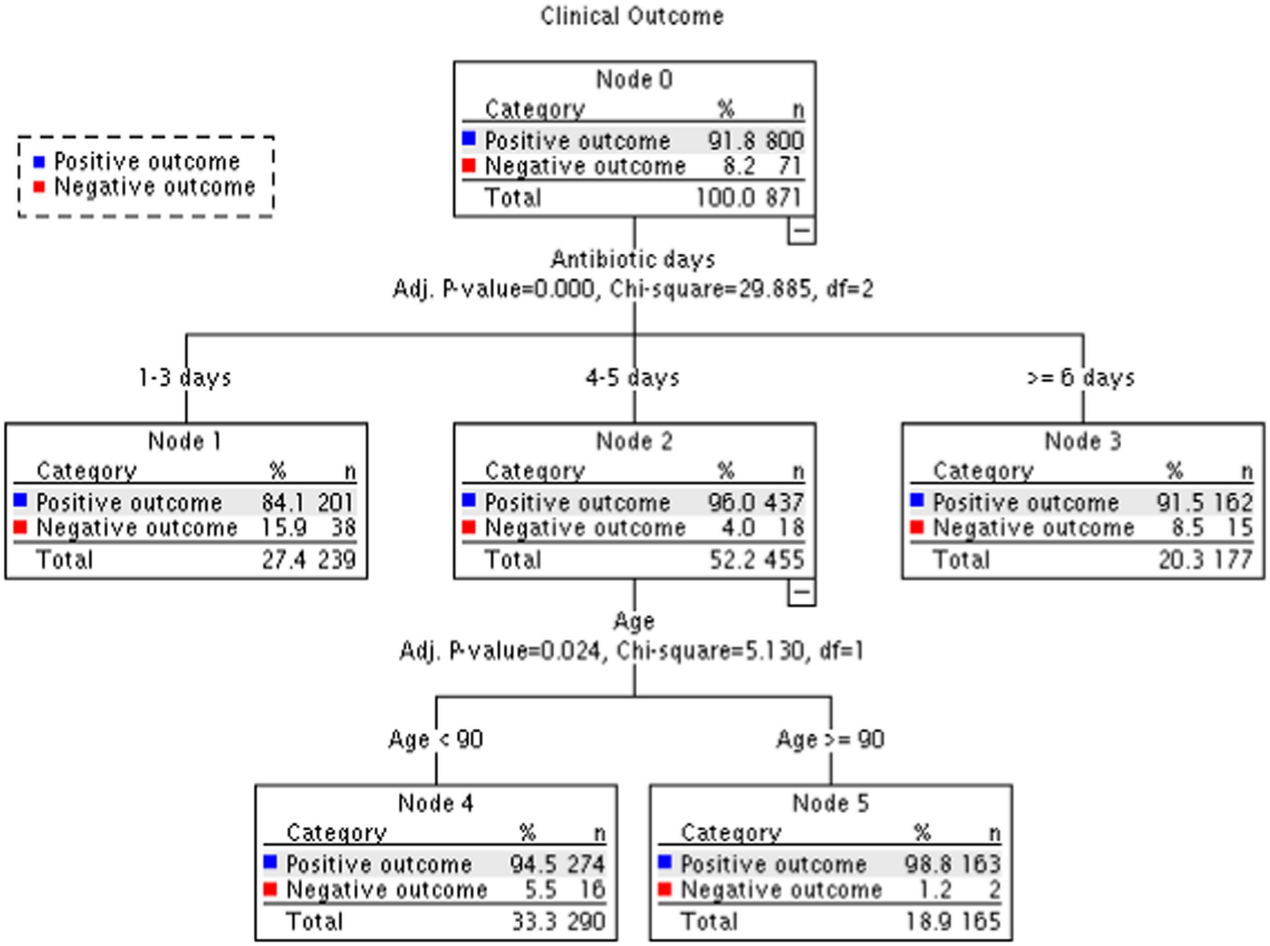
Decision tree for classification patients according to recovery.

Distribution of categorical variables among type of infections as well as the association between these variables with outcome (recovery or negative outcome) were assessed using Chi square test or Fishers exact test in case of 2*2 tables (Tables 1, 2). T-test was used to compare mean age between men and women. We used univariate and multivariable Logistic regression to assess association between factors (diagnosis, patient sex and age, antibiotic type, days of antibiotic treatment, NH type and year of treatment) and recovery (Figure 1; Supplementary Table 1). Collinearity diagnosis shows VIF (variance inflation factor) values <1.4 except for Pneumonia 2.07 and UTI 2.16. We also classified patients according to recovery using a decision tree with CHAID (Chi-squared Automatic Interaction Detection) as a growing method (Figure 2). We performed all the statistical analysis using the IBM SPSS Statistics for Windows, Version 27.0. Armonk, NY: IBM Corp. Two-sided *p*-value ≤0.05 was considered statistically significant.

**Table 1:** Demographic and clinical characteristics of cases by types of infections.

	Total		Pneumonia		UTI		Fever		COPD		*p*
	N	%	N	%	N	%	N	%	N	%	
Total	871	100.0	354	100.0	311	100.0	149	100.0	57	100.0	
Sex											<0.001
Men	342	39.3	123	34.7	151	48.6	51	34.2	17	29.8	
Women	529	60.7	231	65.3	160	51.4	98	65.8	40	70.2	
Age (years)											0.624
< 90	568	65.2	222	62.7	209	67.2	100	67.1	37	64.9	
≥90	303	34.8	132	37.3	102	32.8	49	32.9	20	35.1	
Antibiotic											<0.001
Ceftriaxone	765	87.8	338	95.5	248	79.7	133	89.3	46	80.7	
Amikacin	80	9.2	8	2.3	52	16.7	10	6.7	10	17.5	
Other	26	3.0	8	2.3	11	3.5	6	4.0	1	1.8	
Antibiotic (days)											0.031
1–3	239	27.4	101	28.5	87	28.0	41	27.5	10	17.5	
4–5	455	52.2	184	52.0	174	55.9	68	45.6	29	50.9	
≥6	177	20.3	69	19.5	50	16.1	40	26.8	18	31.6	
Nursing homes											0.002
< 60 cases	148	17.0	56	15.8	52	16.7	38	25.5	2	3.5	
> 140 cases	723	83.0	298	84.2	259	83.3	111	74.5	55	96.5	
Treatment year											<0.001
2012	42	4.8	17	4.8	14	4.5	4	2.7	7	12.3	
2013	243	27.9	94	26.6	116	37.3	22	14.8	11	19.3	
2014	227	26.1	87	24.6	89	28.6	36	24.2	15	26.3	
2015	281	32.3	130	36.7	67	21.5	66	44.3	18	31.6	
2016	78	9.0	26	7.3	25	8.0	21	14.1	6	10.5	

UTI, urinary tract infection; COPD, chronic obstructive pulmonary disease.

**Table 2:** Demographic and clinical characteristics by outcomes.

	Recovery		Negative outcome		Hospitalization		Death		*p* ^*^
	N	%	N	%	N	%	N	%	
Total	800	91.8	71	8.2	62	7.1	9	1.0	
Diagnosis									0.158
Pneumonia	318	89.8	36	10.2	30	8.5	6	1.7	
UTI	293	94.2	18	5.8	16	5.1	2	0.6	
Fever	135	90.6	14	9.4	13	8.7	1	0.7	
COPD	54	94.7	3	5.3	3	5.3	0	0.0	
Sex									0.129
Men	308	90.1	34	9.9	28	8.2	6	1.8	
Women	492	93.0	37	7.0	34	6.4	3	0.6	
Age (years)									<0.001
< 90	508	89.4	60	10.6	54	9.5	6	1.1	
≥90	292	96.4	11	3.6	8	2.6	3	1.0	
Antibiotic									0.551
Ceftriaxone	700	91.5	65	8.5	57	7.5	8	1.0	
Amikacin	76	95.0	4	5.0	3	3.8	1	1.3	
Other	24	92.3	2	7.7	2	7.7	0	0.0	
Antibiotic (days)									<0.001
1–3	201	84.1	38	15.9	36	15.1	2	0.8	
4–5	437	96.0	18	4.0	14	3.1	4	0.9	
≥6	162	91.5	15	8.5	12	6.8	3	1.7	
Nursing homes									0.190
< 60 cases	132	89.2	16	10.8	13	8.8	3	2.0	
> 140 cases	668	92.4	55	7.6	49	6.8	6	0.8	
Treatment year									0.058
2012	34	81.0	8	19.0	6	14.3	2	4.8	
2013	225	92.6	18	7.4	16	6.6	2	0.8	
2014	210	92.5	17	7.5	14	6.2	3	1.3	
2015	256	91.1	25	8.9	24	8.5	1	0.4	
2016	75	96.2	3	3.8	2	2.6	1	1.3	

*p-values were calculated between recovery and negative outcome. UTI, urinary tract infection; COPD, chronic obstructive pulmonary disease.

## Results

**Figure 3:**
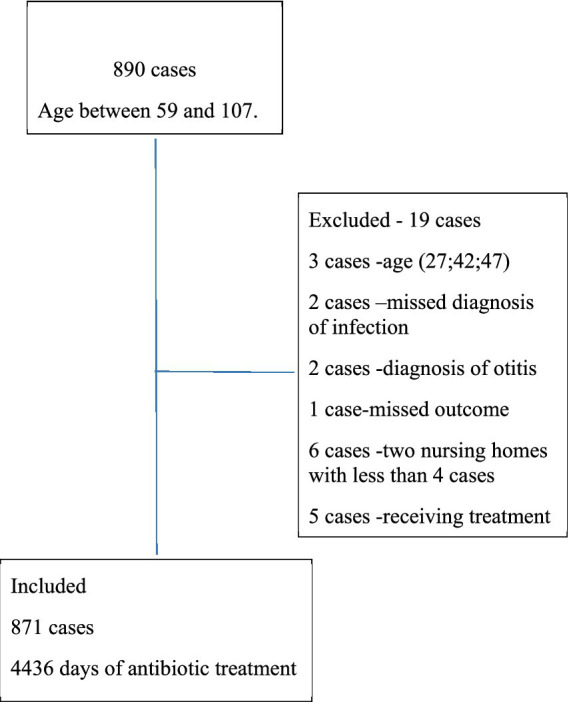
Description of included cases.

Between the 2012–2016, a total of 890 cases with acute infections received IV antibiotics treatment in 10 NH. A total of 871 cases were included in the analysis representing a total of 4,436 treatment days as specified in Figure 3. Demographic and clinical characteristics of the cases are presented in Table 1. The average age was 85.1 ± 7.0 years among men and 86.4 ± 7.8 years among women (*p* = 0.008), the majority (60.7%) of cases were female. Respiratory tract infections were the most common type of infection in our study, with 354 cases (40.6%) diagnosed as acute pneumonia, and 57 cases (6.5%) with Chronic Obstructive Pulmonary Disease (COPD) exacerbation. Additionally, 35.7% of the cases were diagnosed with urinary tract infections (UTI) and 17.1% were listed as fever due to suspected bacterial infection. There were significant differences between men and women in the types of infections, with a higher proportion of women suffering from respiratory tract infections (65.3% of pneumonia cases and 70.2% of COPD cases were women vs. 34.7 and 29.8%, respectively, in men, *p* = 4.4E-04). Significantly more cases with COPD received treatment with antibiotics for more than 6 days (*p* = 0.031). The majority of patients, 765 (87.8%) were treated with IV Ceftriaxone, 80 (9.2%) received aminoglycosides, and 26 cases (3%) were treated with another antibiotic’s regiments or combinations of antibiotics. There was a significant correlation between the type of infection and the antibiotic treatment. Use of aminoglycosides was more prevalent in UTI (16.7%) and COPD (17.5%) vs. pneumonia (2.3%) and fever (6.7%; *p* < 0.001).

Over the course of 4 years, antibiotic treatment was administered to more than 100 cases in most nursing home. Among this cases, 148 cases (17%) were from NH with fewer than 60 treated cases, while 723 cases (83%) were attributed to NH with more than 140 cases treated. The mean number of treatment days was 5.09 ± 3.86, median 5 days (IQR 3–5 days). Approximately 239 cases (27.4%) received treatment for up to 3 days, and 455 cases (52.2%) received treatment for 4 to 5 days and 177 cases (20.3%) received treatment for 6 days or more.

Over one third of the cases, totaling 303 cases (34.8%) were aged 90 years or older, while 568 cases (65.2%) were younger than 90. There was no significant difference between cases younger and older than 90 years in terms of infection type, antibiotic treatment and duration of treatment.

Table 2 presents the outcomes of the intervention program. Of the cases, 800 (91.8%) recovered after receiving in-house IV treatment, while 62 cases (7.1%) required hospitalization in a general care hospital and 9 cases (1.0%) resulted in mortality. There was no significant difference in recovery rates between men and women, different NH, different types of antibiotics. Recovery was seen in 94.2% of UTI cases, 94.7% of COPD cases, 89.8% of pneumonia and 90.6% in cases with fever. However, the differences in recovery rates were not statistically significant.

Cases aged 90 years and older exhibited better recovery rates compared to younger patients (OR = 3.02; 95%CI 1.53–5.95; *p* = 0.001). These differences were mostly driven from a lower hospitalization rate (2.6% vs. 9.5%). Days of treatment were strongly associated with recovery rate. The OR = 4.36 (95%CI 2.40–7.95) and OR = 2.14 (95%CI 1.10–4.19) respectively for 4–5 days and ≥ 6 days compared to treatment duration of 1–3 days (Figure 1; Supplementary Table 1).

Similar results were obtained from the decision tree analysis (Figure 2), which revealed a significant difference between days of treatment and clinical outcomes. In the group treated for 1–3 days, 84.1% of cases recovered, while for 4–5 days of treatment, 96.0% recovered, and in the group treated for 6 days or more, 91.5% recovered (*p* < 0.001). Patients who received antibiotic treatment for 4–5 days exhibited better recovery rates and lower hospitalization rates (nodes 1, 2 and 3). The rates of recovery decline mildly after 5 days of treatment, with the lowest rate observed in the group treated for up to 3 days (positive outcome for 96.0, 91.5 and 84.1% accordingly). Patients aged over 90 years old demonstrated better outcomes with a higher success rate (nodes 4 and 5).

There was no significant difference in outcomes observed among different types of infections.

## Discussion

Over the course of our four-year study, focused on increasing in-house antibiotic treatment in nursing home patients. We presented a unique intervention program that effectively shifted perception of possibility for giving appropriate treatment for acute infections within NH. As a result, 10 NH adopted the practice of treating eligible patients “in-house.” Most of the patients who received in-house antibiotic treatment recovered. Only 8.2% required hospital transfer after starting IV antibiotics. The majority of the patients were successfully treated with single antibiotics and did not require combination regimens or extremely broad antibiotics, which further reinforces the view that quality treatment can be given “in-house.” We observed a learning effect, particularly in the first year where the percentage of negative outcomes was higher. Interestingly, among nursing home residents aged over 90 years old, who are considered to be predisposed to complications compared to younger old patients, the highest recovery rates were observed. This finding may be partly attributed to a reduced inclination to resort to hospitalizations for the oldest old individuals.

Our outcomes support the importance of teaching and administrative interventions to decrease hospital transfers of nursing care patients with acute infections. As opposed to most guidelines which focused on evaluating patients and determining when to refer to acute care (20, 21), our approach focused on increasing awareness and knowledge among nursing home staff and offering support for their clinical decisions.

While hospital care is crucial in certain situations, there are well-known potential risks and complications, particularly for the frail older patients with multiple comorbidities (21, 22). Longer hospital stays, cognitive decline, decubitus ulcers, falls, infections and functional decline have been described in many studies, mostly, among nursing care residents who are particularly vulnerable (23). Ahearn et al. reported a mortality rate of 33.9% among nursing home residents during hospitalization in a general hospital, with a 51.6% mortality rate among at 6 weeks following hospitalization (15).

Studies looking at the appropriateness of referrals of residents from nursing facilities to general hospitals have shown high percentages of unnecessary referrals (10, 24, 25). Saliba et al. reported that 36% of emergency room referrals and 40% of hospital referrals were inappropriate (10). Both quantitative and qualitative studies have analyzed the characteristics and associated factors of inappropriate referrals, suggesting interventions aimed at reducing potentially preventable hospitalizations (25–27). In 2001, practice guidelines were published in the US for treating fever and infections in nursing home residents. These guidelines recommend minimizing hospital referrals and specify in what situations referral to hospital can be avoided (28). One such situation is an acute infection with stable hemodynamic and respiratory measurements (29). Our study focused on increasing in-house antibiotic treatment in appropriate patients. We referenced two previous studies conducted over 15 years ago, both comparing antibiotic treatment in nursing home residents with and without hospital transfer. Dosa et al. compared nursing home residents with acute pneumonia had significantly higher mortality and functional decline compared to those treated in-house (30). Boockvar et al. in the 2005 study found significantly higher mortality and decubitus ulcers in the hospitalized patients with pneumonia compared to those treated in the nursing facility (13). The spectrum of infections in our study, primarily involving lower respiratory tract and urinary tract infections, was similar to that reported in other countries (31). Despite our patients belonging to the highest risk category as residents of NH, our outcomes were better than those of older patients admitted to hospitals with pneumonia (32) and mortality rate was lower (1%) than those reported in the literature for NH residents (33).

In our study, there was a significant cohort of nursing home residents aged over 90. Previous studies have often indicated higher mortality rates among of patients older than 85, with studies like Lee et al. (34) demonstrating that octogenarians had the highest mortality rates, and Garibaldi et all (35) suggesting that older adult patients are predisposed to serious infections due to underlying chronic or acute illness. As opposite to these findings, our study revealed that the group of patients older than 90 exhibited the highest recovery rates. This finding carries potential clinical implications and may impact decisions regarding hospitalization indications for octogenarians.

Our study has several limitations. We focused on outcomes post- intervention in 10 NH lacking pre-intervention data for comparison. Advanced health care directives and patient preferences were not assessed and may theoretically influence hospitalization rate. Giving the high recovery rate, there is a low probability for significant selection bias. We also did not include a comparison with NH that did not receive an intervention, limiting our ability to gage the intervention’s effectiveness comprehensively. We did not obtain bacterial culture as part of the protocol, potentially impacting the quality of care, mainly for UTIs. However, we believed making cultures mandatory may have lowered compliance from the stuff. Primary diagnosis and treatment are based on clinical evaluation, given the common occurrence of asymptomatic bacteriuria in NH (36). We looked at the three parts of the intervention as a whole, and therefore, cannot say which part was the most effective—the educational aspect, the clinical support, or the logistic intervention. Moreover, while we did not assess costs or cost-effectiveness, our interventions used resources within the HMO, and therefore were low cost. Nonetheless, they effectively prevented costly outcomes such as hospitalizations and deaths (37, 38), highlighting their value both economically and in terms of human life.

## Conclusion

Our study provides an example of an effective intervention for improving outcomes of acute infections in nursing home residents. By focusing on increasing awareness and knowledge among nursing home staff, and facilitating the logistics of in-house IV antibiotic treatment, it is possible to improve compliance, achieved high recovery rates and minimized hospitalizations, thus improving quality of care in NH residents.

## Data availability statement

The data analyzed in this study is subject to the following licenses/restrictions: data set is from classified EMR not open to the public. Requests to access these datasets should be directed to batia_nadya.kagansky@moh.gov.il.

## Ethics statement

The studies involving humans were approved by the Kupat Cholim Clalit Central ethics committee and conforms to the principles outlined in the Declaration of Helsinki (IRB 0234-16-COM2). Due to the study’s retrospective nature, informed consent was wavered by the ethics committee. The studies were conducted in accordance with the local legislation and institutional requirements. Written informed consent for participation was not required from the participants or the participants’ legal guardians/next of kin in accordance with the national legislation and institutional requirements.

## Author contributions

NK: Conceptualization, Supervision, Data curation, Investigation, Methodology, Writing – original draft. RR: Data curation, Investigation, Resources, Writing – review & editing. ED: Writing – review & editing, Formal analysis, Methodology, Visualization. EM: Writing – review & editing, Investigation, Validation. YL: Writing – review & editing, Conceptualization, Project administration, Supervision, Visualization. MB: Supervision, Validation, Writing – review & editing.
