# Supplementary material for: Implementation of a program for treatment of acute infections in nursing homes without hospital transfer
Source: Front Med (Lausanne). 2024 May 20;11:1333523. doi: 10.3389/fmed.2024.1333523 (PMC11144856; doi:10.3389/fmed.2024.1333523)
Supplement: Supplementary file 1 [file Table_1.DOCX]

|  | **Recovery** | |  | | | |
| --- | --- | --- | --- | --- | --- | --- |
|  | **N** | **%** | **B** | **S.E.** | **OR (95%CI)** | **P** |
| **Age ≥90 years** | 292 | 96.4 | 1.10 | 0.35 | 3.02 (1.53-5.95) | 0.001 |
| **Antibiotic (days)** |  |  |  |  |  | 7.8E-06 |
| 1-3 | 201 | 84.1 |  |  | 1 |  |
| 4-5 | 437 | 96.0 | 1.47 | 0.31 | 4.36 (2.4-7.95) | 1.6E-06 |
| ≥6 | 162 | 91.5 | 0.76 | 0.34 | 2.14 (1.10-4.19) | 0.025 |
| **Women** | 492 | 93.0 | 0.37 | 0.27 | 1.44 (.86-2.43) | 0.171 |
| **Large institute** | 668 | 92.4 | 0.40 | 0.33 | 1.50 (0.79-2.84) | 0.214 |
| **Diagnosis** |  |  |  |  |  | .165 |
| Pneumonia | 318 | 89.8 |  |  | 1 |  |
| UTI | 293 | 94.2 | 0.68 | 0.32 | 1.97 (1.06-3.65) | .032 |
| Fever | 135 | 90.6 | 0.20 | 0.35 | 1.22 (0.62-2.42) | .568 |
| COPD | 54 | 94.7 | 0.66 | 0.64 | 1.94 (0.55-6.85) | .302 |
| **Treatment year** |  |  |  |  |  | .140 |
| 2012 | 34 | 81.0 | -1.60 | 0.74 | 0.20 (0.05-0.87) | .032 |
| 2013 | 225 | 92.6 | -0.48 | 0.66 | 0.62 (0.17-2.26) | .467 |
| 2014 | 210 | 92.5 | -0.77 | 0.66 | 0.46 (0.13-1.70) | .245 |
| 2015 | 256 | 91.1 | -0.87 | 0.64 | 0.42 (0.12-1.48) | .177 |
| 2016 | 75 | 96.2 |  |  | 1 |  |

**Supplement**

**Table*** Variables included in the model: diagnosis, patient sex and age, antibiotic type, days of antibiotic treatment, nursing homes type and year of treatment. Variables not statistically

* Variables included in the model: diagnosis, patient sex and age, antibiotic type, days of antibiotic treatment, nursing homes type and year of treatment. Variables not statistically significant were not included in figure.

Abbreviations: UTI-urinary tract infection, COPD-chronic obstructive pulmonary disease, OR-Odds Ratio
